# Bioelectrical Impedance Analysis (BIA) of the association of the Japanese Kampo concept “Suidoku” (fluid disturbance) and the body composition of women

**DOI:** 10.1186/s12906-016-1373-9

**Published:** 2016-10-22

**Authors:** Aya Murakami, Daisuke Kobayashi, Toshio Kubota, Niina Zukeyama, Haru Mukae, Norihiro Furusyo, Mosaburo Kainuma, Takao Shimazoe

**Affiliations:** 1 Department of Clinical Pharmacy and Pharmaceutical Care, Graduate School of Pharmaceutical Sciences, Kyushu University, 3-1-1 Maidashi, Higashi-ku, Fukuoka, 812-8582 Japan; 2 Community Medicine Education Unit, Graduate School of Medical Science, Kyushu University, 3-1-1 Maidashi, Higashi-ku, Fukuoka, 812-8582 Japan; 3 Department of General Internal Medicine, Kyushu University, 3-1-1 Maidashi, Higashi-ku, Fukuoka, 812-8582 Japan

**Keywords:** Suidoku, Fluid disturbance, Bioelectrical impedance analysis, Body composition, Kampo

## Abstract

**Background:**

In Japanese Kampo medical practice, suidoku (fluid disturbance) is one of the most important concepts for selecting the proper medication. Suidoku is an excessive or uneven distribution of fluid that is indicated by splashing sounds and pitting edema. However, few objective reports about suidoku have been published. Bioelectrical impedance analysis (BIA) uses resistance values obtained from weak electrical currents to estimate body composition, including intracellular and extracellular water and muscle and fat mass. In this study, we used BIA to search for objective factors that can discriminate the various types of suidoku.

**Methods:**

Two hundred twenty-nine patients who visited the Kampo Medicine Clinic of Kyushu University Hospital from June 2010 to August 2015 were divided into non-suidoku (*n* = 180, 80 male and 100 female), splashing sound (*n* = 32, 8 male and 24 female) and edema groups (*n* = 17, 5 male and 12 female). Body composition values were taken from the electronic medical records of InBody730 (a vertical, segmental, multi-frequency analyzer by InBody, Tokyo Japan) testing done at the initial visit. Various parameters of the body composition values of female in the non-suidoku and suidoku groups (splashing sound and edema groups) were compared: there were too few male patients to provide significance.

**Results:**

The age and body weight were significantly lower in the splashing sound group than in the non-suidoku group (*p* < 0.05). In contrast, the body weight of the edema group was significantly heavier than that of the non-suidoku group (*p* < 0.05). In ROC analysis, the percent Body Fat ≤ 27.8 %, Muscle Mass Index of the Trunk ≤ 6.5 kg/m^2^, VFA (Visceral fat area) ≤ 5.4 and BMI ≤ 19.2 kg/m^2^ were associated with splashing sound, and Muscle Mass Index of Legs ≥ 4.8 kg/m^2^ and BMI ≥ 21.4 kg/m^2^ were associated with edema.

**Conclusion:**

Our data suggest that the use of this type of BIA to estimate body composition would be a useful tool for the diagnosis of suidoku for women.

## Background

Traditional Chinese medicine as brought from ancient China to Japan has been modified over the centuries to its current form as traditional Japanese medicine (Kampo). Kampo is widely used in clinical practice, with about 90 % of the physicians and almost 100 % of Japanese obstetrics and gynecology specialists in Japan using it in their daily practice [1–3].

Kampo diagnosis includes the basic concepts “Yin-Sho and Yo-Sho (chilly, inactive, or inhibitory and feverish, active or excitatory, respectively)”, “Jitsu-Sho and Kyo-Sho (strong and weak)”, “Ki, Ketsu and Sui”, “Roku-Byoui (six disease phase)”, and “Gozo (five organs)”. “Ki, Ketsu and Sui” are thought of as the three major elements necessary for life [4, 5]. “Ki” can be understood as an intangible energy fundamental to living things. In contrast to “Ki”, “Ketsu” and “Sui” are more material in nature and probably much closer to the usual concepts of blood and body fluids, respectively. “Ketsu” is understood as the blood that gives nutrition to the internal organs as it flows through the body along with “Ki”. “Sui” is understood as being colorless liquids other than blood, such as lymph fluid. Illness is considered to be the result of an imbalanced state of Ki, Ketsu and Sui, and a Kampo diagnosis is referred to as “Sho”. Suidoku, fluid disturbance, is an aspect of Sho that refers to an imbalance of Sui (excessive or uneven distribution). Typical subjective symptoms of suidoku are dizziness, swelling, diarrhea, and stiffness of the body, and the objective symptoms include splashing sounds, pitting edema, swollen tongue and dental indentation.

Kampo medicines are prescribed according to the Sho diagnosis; therefore, an accurate Sho diagnosis leads to the proper use of Kampo medicines and to the best result for the patient. A weakness of Sho diagnosis is that it depends on subjective factors such as the knowledge and experience of the practitioner. Some reports have pointed out that diagnoses are often inconsistent and lack objectivity [6–8]. Recently, many studies have been done to gain objective evaluations of the therapeutic effects of Kampo medicines [9, 10] and to develop objective indexes for making a diagnosis [11–16]. Few studies have been done to objectively evaluate suidoku [17, 18].

Bioelectrical impedance analysis (BIA) uses resistance values (impedance) obtained from weak electrical currents to estimate body composition, including the intracellular and extracellular water percentages and muscle and fat mass percentages [19, 20]. It is known that BIA can estimate the water balance as Extracellular Water / Total Body Water (ECW/TBW) [21]. BIA is non-invasive, can estimate in only a few minutes, and does not need to take into account racial differences [22]. For these reasons, we choose BIA to objectively evaluate suidoku. Body composition analyzers have been used in various fields of research, such as gastrointestinal medicine, renal dialysis and diabetes mellitus, with aims that ranged from understanding pathological conditions to predicting the clinical outcome after operations [23–26].

The aim of this study was to use BIA to search for objective factors that can discriminate the various types of suidoku found in a cohort of patients who had previously been diagnosed with suidoku. Questionnaires were used to gather data on subjective symptoms. The full cohort was subdivided into splashing sound and edema groups based on their diagnosis. In order to determine the factors related to suidoku, various parameters of body composition and the subjective symptoms of the suidoku and non-suidoku groups were compared and assessed by the cut-off value from ROC analysis.

## Method

### Patient enrollment

Retrospective analysis was done of the data of 557 new patients (≥20 years) who visited the Kampo Medicine Clinic of Kyushu University Hospital from June 2010 to August 2015. The exclusion criteria included patients taking Kampo medicines, an antivertigo medicine, or diuretics; patients for whom electronic medical records were not available and patients who subjectively reported swelling from edema at their initial visit, but for whom edema was not found when objective testing was done by their doctor. Although, it is known that a swollen tongue and dental indentation are objective indications of suidoku, we excluded patients with these conditions because the data in the medical records was insufficient. The patients were divided into a suidoku group and a non-suidoku group based on their electronic medical records. The suidoku group was divided into a splashing sound group, confirmed by physical examination, and an edema group with pretibial pitting edema. A splashing sound can be heard over the epigastric region or the third portion of the duodenum or jejunum on auscultation when tapping with a flexible wrist. This finding indicates reduced abdominal tension in this area, air in the stomach, and fluid retention in stomach, duodenum, or jejunum. Pitting edema can be demonstrated by applying pressure to a swollen area by depressing the skin with a finger. If an indentation remains after the release of pressure after pressing for about 10 s, the edema is referred to as pitting edema. These tests are invariably performed to ascertain the presence of findings in Kampo medicine. The study was conducted in accordance with the principles of the Helsinki Declaration of 1975, as revised in 2000.

### Analysis of body composition

Body composition values were taken from the electronic medical records of InBody730 (InBody, Tokyo Japan) testing done at the initial visit. InBody730 is a body composition analyzer that estimates segmental body composition (arms, trunk and legs) using multiple frequencies (1, 5, 50, 250, 500 and 1000 kHz). The BIA analyzer we used can display Extracellular Water / Total Body Water as the water balance. The patients were instructed to grasp the handles of the analyzer in which electrodes are embedded and to stand on electrodes that contact the bottoms of their feet (two electrodes for each foot and hand). All new patients are tested with InBody730 before diagnosis in our Kampo clinic. The items reported are Percent Body Fat, Extracellular Water / Total Body Water (ECW/TBW) (Arms, Trunk, and Legs), VFA (Visceral Fat Area), Muscle Mass Index (Arms, Trunk and Legs) and BMI. Muscle Mass Index is expressed as muscle weight divided by the square of body height (kg/m^2^). VFA is the visceral fat area (cm^2^), with 10 indicating a visceral fat area of 100 (cm^2^). The body composition values of the non-suidoku group were compared with those of the splashing sound and edema groups.

### Analysis of subjective symptoms

A questionnaire was used to determine the relation between subjective symptoms and suidoku. The questionnaire consisted of 231 questions, including the physical condition of the patients. This questionnaire included eight items related to suidoku: “Head feels heavy as if covered by something”, “Frequent dizziness”, “Frequent runny nose”, “Sometimes giddy”, “Sometimes have swelling”, “Headache dependent upon the weather”, “Feel sluggish” and “Hands, joints, or body sometimes feel stiff in the morning”. The patients were asked to answer all questions using a five point scale: 0, none, 1 slight, 2 mild, 3 moderate and 4 severe, with 0 or 1 considered to have “no subjective symptoms” and 2, 3 and 4 considered to “have subjective symptoms”. The answers of the non-suidoku group were compared with those of the splashing sound and edema groups.

### Statistical analysis

Ratios were compared using the chi-square test. Results of the body composition analysis are expressed as the mean ± standard deviation. Height, body weight and age were compared using Wilcoxon’s rank sum test. The body composition (9 items) and subjective symptom analyses were compared to the non-suidoku group using logistic regression analysis, and odds ratio (OR), 95 % confidence interval (95 % CI) and p-value were calculated. ROC analysis was used to calculate cut-off values, sensitivity (Se), specificity (Sp) and area under the curve (AUC). All analyses were conducted using JMP ver.11 software and *p* < 0.05 was considered significant.

## Results

### Participant selection

**Fig. 1:**
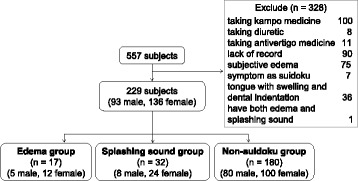
Participant Selection. The patients were divided into three groups according to their diagnosis at the initial visit. The exclusion criteria included patients currently taking a Kampo medicine, antivertigo medicine, or diuretics; those for whom electronic medical records were not available; and those with subjective edema, swollen tongue, or dental indentation

Of the 557 patients, 328 were excluded, leaving the data of 229 available for analysis (Fig. 1). The patients were divided into a non-suidoku group (*n* = 180, 80 male and 100 female), a splashing sound group (*n* = 32, 8 male and 24 female) and an edema group (*n* = 17, 5 male and 12 female). A significantly higher number of female than male patients was found in the splashing sound group (OR: 2.40, 95%CI: 1.02–5.63, *p* < 0.05). There were more female than male patients in the edema group, but the difference did not reach significance (OR: 1.92, 95 % CI: 0.65–5.68, *p* = 0.17). Because there were too few male patients to give significance to the results, our analysis of body composition and subjective symptoms was done only with female patients.

### Clinical characteristics and body composition parameters

The data on the body composition of the female patients is shown in Table 1. The splashing sound group was, on average, 13.7 years younger than the non-suidoku group, and they were 5.5 cm taller and 3.4 kg of body weight lower. In contrast, the body weight in the edema group was 7.3 kg heavier than that of the non-suidoku group.

**Table 1:** Characteristics of subjects and body composition parameter using body composition analyzer

	Non-suidoku group (*n* = 100)	Suidoku group	
		Splashing sound group (*n* = 24)	Edema group (*n* = 12)
Age (years)	59.8 ± 14.0	46.1 ± 18.1 ^*^	59.5 ± 18.4
Height (cm)	153.6 ± 5.7	159.1 ± 8.0 ^*^	154.9 ± 6.4
Body weight (kg)	50.8 ± 7.8	47.4 ± 5.7 ^*^	58.1 ± 9.6 ^*^
Percent body fat (%)	30.8 ± 6.9	24.9 ± 5.2	34.6 ± 6.9
ECW/TBW			
Arms	0.381 ± 0.004	0.380 ± 0.004	0.382 ± 0.004
Trunk	0.391 ± 0.006	0.390 ± 0.006	0.392 ± 0.006
Legs	0.393 ± 0.007	0.392 ± 0.008	0.395 ± 0.008
Muscle Mass Index (kg/m^2^)			
Arms	1.3 ± 0.2	1.2 ± 0.2	1.4 ± 0.2
Trunk	6.5 ± 0.7	6.0 ± 0.5	6.8 ± 0.6
Legs	4.4 ± 0.5	4.4 ± 0.5	4.8 ± 0.6
VFA	9.2 ± 3.0	5.7 ± 2.9	10.3 ± 3.6
BMI (kg/m^2^)	21.6 ± 3.4	18.7 ± 1.9	24.2 ± 3.5

mean ± S.D

*ECW*/*TBW* Extracellular Water / Total body Water, *VFA* visceral fat area, *BMI* body weight divided by the square of body height (kg/m2)

*p < 0.05 vs non-suidoku group, Wilcoxon rank sum test. Performed at Age, Height and Body weight

### Analysis of body composition

Logistic regression analysis identified five factors that were significantly lower in the splashing sound group: Percent Body Fat, Muscle Mass Index of the Arms, Muscle Mass Index of the Trunk, VFA and BMI. In the edema group, two factors were significantly higher than in the non-suidoku group: Muscle Mass Index of the Legs and BMI (Table 2).

**Table 2:** Predictors of body composition for the diagnosis of suidoku

	Splashing sound group (*n* = 24)			Edema group (*n* = 12)		
	OR	95 % CI	*P*	OR	95 % CI	*P*
Percent body fat (%)	0.84	0.76–0.91	< 0.01	1.07	0.99–1.17	0.086
ECW/TBW						
Arms	0.20	0.02–1.51	0.120	1.77	0.12–27.5	0.677
Trunk	0.52	0.05–5.06	0.574	2.41	0.11–51.9	0.575
Legs	0.40	0.04–4.00	0.434	4.09	0.18–96.5	0.373
Muscle Mass Index (kg/m^2^)						
Arms	0.06	0.01–0.51	< 0.01	8.85	0.70–119	0.092
Trunk	0.29	0.12–0.63	< 0.01	1.90	0.81–4.50	0.140
Legs	1.07	0.41–2.91	0.890	6.71	1.75–32.1	< 0.01
VFA	0.66	0.54–0.79	< 0.01	1.12	0.93–1.36	0.226
BMI (kg/m^2^)	0.61	0.45–0.78	< 0.01	1.18	1.02–1.38	0.03

*OR* odds ratio, *95* % *CI* confidence interval, *ECW*/*EBW* Extracellular Water / Total body Water, *VFA* visceral fat area, *BMI* body weight divided by the square of body height (kg/m^2^)

**Fig. 2:**
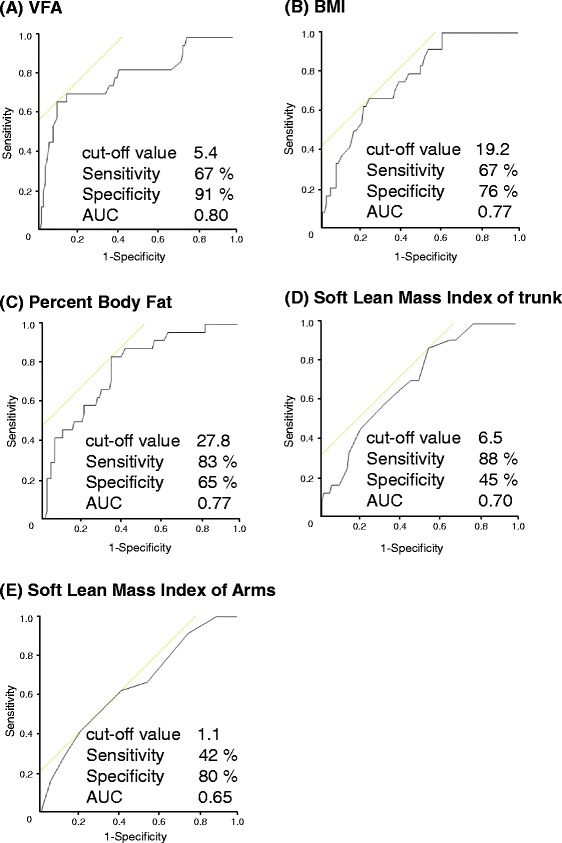
Cut-off values for the discrimination of splashing sound (ROC curve). **a** VFA, **b** BMI, **c** Percent Body Fat, **d** Muscle Mass Index of the trunk, **e** Muscle Mass Index of the Arms. Cut-off values for the estimation of suidoku were calculated using ROC. In the splashing sound group, the cut-off values were VFA 5.4 (Se: 67 %, Sp: 91 %, AUC: 0.80), BMI 19.2 kg/m^2^ (Se: 67 %, Sp: 76 %, AUC: 0.77), Percent Body Fat 27.8 % (Se: 83 %, Sp: 65 %, AUC: 0.77), Muscle Mass Index of the Trunk 6.5 kg/m^2^ (Se: 88 %, Sp: 45 %, AUC: 0.70), and Muscle Mass Index of the Arms 1.1 kg/m^2^ (Se: 42 %, Sp: 80 %, AUC: 0.65)

**Fig. 3:**
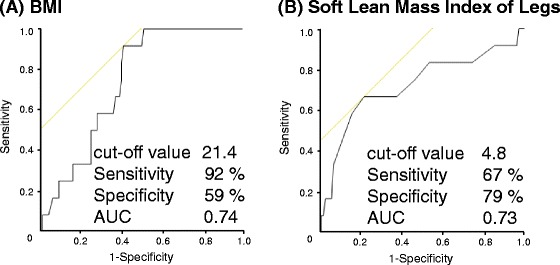
Cut-off values for the discrimination of edema (ROC curve). **a** BMI, **b** Muscle Mass Index of the Legs. Cut-off values were BMI 21.4 kg/m^2^ (Se: 92 %, Sp: 59 %, AUC: 0.74) and Muscle Mass Index of the Legs 4.8 kg/m^2^ (Se: 67 %, Sp: 79 %, AUC: 0.73)

Cut-off values for the estimation of suidoku were calculated using ROC. In the splashing sound group, the cut-off values were VFA 5.4, BMI 19.2 kg/m^2^, Percent Body Fat 27.8 %, Muscle Mass Index of the Trunk 6.5 kg/m^2^ and Muscle Mass Index of the Arms 1.1 kg/m^2^ (Fig. 2). In the edema group, the cut-off values were BMI 21.4 kg/m^2^ and Muscle Mass Index of the Legs 4.8 kg/m^2^ (Fig. 3).

### Analysis of subjective symptoms

From the questionnaire, eight subjective symptoms were extracted for the splashing sound group and one for the edema group. In the splashing sound group, the most common subjective symptom was “hands and legs feel cold” at 91.7 %, followed by “Dry skin” at 83.3 % and “Need electric blanket or warmers in winter” at 75.0 % (Table 3). However, three of the eight subjective symptoms that were thought before the study to possibly be related to suidoku, “Frequent runny nose”, “Sometimes swelling”, and “Headache dependent upon the weather”, were significantly higher than in the non-suidoku group; nevertheless, their response rates were less than 50 %. The other four subjective symptoms were not significant. In contrast, “In the morning, sometimes have stiffness in hands, joints, or body” was 58.3 and 20.0 %, respectively, in the edema and non-suidoku groups.

**Table 3:** Subjective symptom related to splashing sound

	OR	95 % CI	*P*	Subjective symptom (%)	
				Non-suidoku group	Splashing sound group
Hands and legs feel cold	7.97	2.18 – 51.4	< 0.01	58.0	91.7
Dry skin	6.90	2.40 – 25.1	< 0.01	42.0	83.3
Need electric blanket or warmers in winter	2.88	1.11 – 8.50	< 0.05	51.0	75.0
There is something stomachache	3.98	1.59 – 10.3	< 0.01	26.0	58.3
Head feels heavy as covered something	3.36	1.34 – 8.59	< 0.01	26.0	54.2
Feel sluggish	1.27	0.52–3.13	0.597	44.0	50.0
Sometimes have swelling	3.61	1.39–9.36	< 0.05	19.0	45.8
Frequent runny nose	3.38	1.31–8.73	< 0.05	20.0	45.8
Headache dependent upon the weather	4.39	1.62–11.9	< 0.01	14.0	41.7
Frequent dizziness	2.13	0.76–5.63	0.141	19.0	33.3
Sometimes giddy	3.02	1.00–8.69	0.050	12.0	29.2
Hands, joints or body sometimes feel stiff in the morning	0.57	0.13–1.87	0.387	20.0	12.5

*OR* odds ratio, *95* % *CI* confidence interval

## Discussion

This is the first study using BIA to demonstrate an association between suidoku and body composition and to identify factors associated with suidoku. Many problems related to a lack of objectivity have been reported for Kampo medicine. Lo et al. showed low rates of agreement in tongue diagnoses, particularly differences in judgment about tongue color, and reported the usefulness of an automated tongue diagnosis system [6]. They also stated that tongue diagnosis is often biased by the diagnostic skill level, experience and color perception. Furthermore, Ishida et al. reported that a skin moisture deficiency might be an indicator of blood deficiency [12]. Although the establishment of objective indices is desired, reports based on objective evaluations of suidoku are still lacking. Although there have been some reports about factors associated with suidoku, they analyzed subjective symptoms. Because no studies have been published that have attempted to objectively quantify suidoku [17, 18], this study was done using a body composition analyzer to identify factors associated with suidoku that would be useful in making a Kanpo diagnosis.

About 60 % of our new patients who presented for treatment with kampo medicines were female, and other studies have similarly reported a high proportion of female patients reporting to Kampo clinics [27, 28]. In our study, the subjective symptom analysis was done only with female patients because of the small number of male patients and in consideration of the difference in physical size.

The logistic regression analysis found significantly lower Percent Body Fat, Muscle Mass Index of the Arms, Muscle Mass Index of the Trunk, VFA and BMI, in the splashing sound group than in the non-suidoku group. Terasawa et al. studied the physiology of the stomach and the amount of gastric juice in a cohort of patients with splashing sounds and reported on the mechanisms and conditions related to the manifestation of splashing sounds [29]. They found that splashing sounds require an appropriate amount of air and gastric juices and that the occurrence of splashing sounds is associated with weak abdominal strength and a drooping of the stomach antrum. It is possible that low VFA indicates gastroptosis because fat supporting the internal organs is low. Also, low Muscle Mass of the trunk, which includes the abdominal muscles, may indicate weak abdominal strength. Thus, our results support a previous finding that splashing sounds occur when oscillation of gastroptosis is done through weak abdominal musculature. Furthermore, many of the patients in our splashing sound group had low BMI or Percent of Body Fat, thus splashing sounds may mainly occur in people who are thin. Some studies have reported that BMI was lower in patients with gastroptosis than in these without gastroptosis subjects and tends to be more common in people who are thin [30], so there is the possibility that people who manifest the splashing sound may tend to have gastroptosis.

By ROC analysis, we identified VFA ≤ 5.4, BMI ≤ 19.2 kg/m^2^, Percent Body Fat ≤ 27.8 %, Muscle Mass Index of the Trunk ≤ 6.5 kg/m^2^, as being associated with splashing sound. Muscle Mass Index of the Arms was not a useful indicator because of the low sensitivity, specificity and AUC (Se: 42 %, Sp: 80 %, AUC: 0.65). On the other hand, BMI and Muscle Mass Index of the Legs might be associated with edema. However the number of patients, 12, in this group was small, thus the data of more patients will have to be gathered for future analysis.

In the analysis of our questionnaire, the percent of participants responding “cold hands and feet” and “Need electric blankets or warmers in winter” was 91.7 and 75.0 %, respectively. Both were significantly high, suggesting the possibility of an association between splashing sound and “cold”. Yamato et al. reported that the sensation of feeling cold is stronger in patients whose body weight, BMI, skinfold thickness, percent body fat and amount of fat are low [31]. They suggested that body weight loss in the form of fat free mass loss, leads to reduced metabolism and reduced blood flow velocity, which induced the sensation of cold. Similarly, Yoshino et al. in a study of the sense of cold and its trends reported that a decrease of heat production by reducing muscle mass results in decreased basal metabolism, which leads to cold sensations [27]. In our study, BMI, percent body fat and the Muscle Mass Index of the Trunk of the splashing sound group was lower than that of the non-suidoku group. The association of a sense of cold and suidoku has been recognized in clinical practice. It is possible that the “cold” symptoms are caused by the distribution of water, which is due to the disruption of water circulation by the decreasing metabolism. Our results support the association of cold and suidoku. Furthermore, 83.3 % of the respondents complained of “dry skin”, which may represent an uneven distribution of water: a lack of water compared to the amount normally present in skin. In Kampo, it is understood that intestinal function affects the skin condition. Some papers have reported that patients have improved skin condition after taking Kampo medicine for gastrointestinal weakness [32, 33]. It is possible that people with splashing sounds trend to have gastroptosis and skin symptoms caused by poor digestion. The improvement of gastrointestinal problems may lead to improved skin condition. In the edema group, we obtained a high response rate for the subjective symptom “In the morning, sometimes have stiffness in hands, joints, or body” (edema group and non-suidoku group 58.3 and 20.0 % respectively). It is said that edema is an uneven distribution of water in stroma, thus stiffness may similarly occur throughout the body. Suidoku primarily signifies an excessive or uneven distribution of water and is understood to represent an irregularity of water balance. In this study, we found that both the splashing sound and edema groups had symptoms related to an uneven distribution of water.

A splashing sound can be heard on palpation of the abdomen, an abdominal diagnosis technique used in Sho diagnosis that was developed in Japan and is unique to Japanese Kampo medicine [34]. It is an important examination technique that provides extremely valuable information for making a Sho diagnosis. It is sometimes viewed with suspicion because it depends on the sensitivity of the doctor's hands and the patient’s reaction to compression of the abdomen. Recently, the development of a simulator to teach palpation of the abdomen and abdominal diagnosis has been proposed. Such a simulator would be desirable as a means of helping standardize sense related factors and improving proficiency [35], but there remain problems with objectivity. Therefore, it is hoped that the indicators of and the questionnaire for suidoku of our study will be helpful to doctors who are short on diagnostic experience.

There were some limitations to our study. First, the number of patients studied was small. Also, we were only able to study female patients, so it will be necessary going forward to perform more detailed research to determine if similar tendencies are observed in male as were observed in the female patients in our study. Second, there was little information on the electronic medical records because it was a retrospective analysis. For a diagnosis of suidoku, both dental indentation and swelling of the tongue are necessary. If the tongue has only dental indentation, it might indicate “Ki-kyo”. Unfortunately, there was too little information on swelling to be of use in this study. We had to exclude 36 patients, including some suspected of suidoku, in order to accurately do an objective assessment. Third, we did not consider the effect of possible confounders. The possibility that BMI may be a confounder cannot be denied. We performed only univariate regression analysis in this study because we judged that multivariate regression analysis would not be useful because of the number of patients and independent variables. Fourth, we did not consider possible differences in the ability of the BIA instrument.

Although InBody730 can display water balance, suidoku has no relevance to water balance. Therefore, we think that the body composition values we extracted in this study are accurate and can be verified by studies using BIA analyzers with similar features to the one used in this study but that do not display water balance. It will be necessary perform future research to address these issues. Furthermore, we discussed a possible association of splashing sound and gastroptosis, but no objective data was obtained for this association, making further study necessary.

## Conclusion

The use of BIA that estimates segmental body composition using multiple frequencies would be a useful tool for the diagnosis of suidoku in Japanese women.

## Abbreviations

95 % CI: 95 % confidence interval
AUC: Area under the curve
OR: Odd ratio
ROC: Receiver Operating Characteristic
Se: Sensitivity
Sp: Specificity
